# PDIA3 correlates with clinical malignant features and immune signature in human gliomas

**DOI:** 10.18632/aging.103601

**Published:** 2020-07-19

**Authors:** Hao Zhang, Yulai Zhou, Quan Cheng, Ziyu Dai, Zeyu Wang, Fangkun Liu, Fan Fan, Biqi Cui, Hui Cao

**Affiliations:** 1 Department of Neurosurgery, Xiangya Hospital, Central South University, Changsha, China; 2 Department of Oncology, Xiangya Hospital, Central South University, Changsha 410008, Hunan, China; 3 National Clinical Research Center for Geriatric Disorders, Xiangya Hospital, Central South University, Changsha 410008, Hunan, China; 4 Department of Clinical Pharmacology, Xiangya Hospital, Central South University, Hunan, China; 5 Department of Neurology, Xiangya Hospital, Central South University, Changsha 410008, Hunan, China; 6 Department of Psychiatry, The Second People’s Hospital of Hunan Province, The Hospital of Hunan University of Chinese Medicine, Changsha, Hunan, China; 7 Equal contribution

**Keywords:** PDIA3, prognosis, tumor microenvironment, immune response

## Abstract

Since therapeutic strategies are limited in gliomas, new molecules or biomarkers are essential for diagnosis and therapy. Here, we investigated expression of protein disulfide isomerase family A member 3 (PDIA3) in gliomas to evaluate its potential as a promising immune target or biomarker. Transcriptome level, genomic profiles and its association with clinical practice from TCGA and CGGA databases were analyzed. All statistical analyses were performed using R project. In gliomas with high PDIA3 expression, somatic mutations showed the correlation with loss of PTEN and amplification of EGFR; meanwhile, in PDIA3 low gliomas, mutations in isocitrate dehydrogenase (IDH) took 80%. Moreover, PDIA3 was found to positively correlate with ESTIMATE scores and diverse infiltrating immune and stromal cell types localizing in tumor microenvironment. PDIA3 was found to be highly correlated with macrophage and T cells based on single cell sequencing. Additionally, PDIA3 was also involved in suppression of anti-tumor immunity via multiple immune regulatory processes. Finally, PDIA3 was observed to correlate with other immune checkpoint inhibitors and associated with inflammation. Our findings identified the significance of PDIA3 in the process of gliomas and demonstrated the potential of PDIA3 as a molecular target in prognosis and immune related treatment of gliomas

## INTRODUCTION

Gliomas are the most common adult primary malignant tumors of the central nervous system (CNS), of which glioblastoma multiform (GBM) is the most malignant subtype [1, 2]. Half of all newly diagnosed patients with glioblastoma are over the age of 65 years in America. Five years after the diagnosis, only 2.4% of the patients aged 65 to 74 and 1.1% of the patients aged 75 or older are alive [3]. Despite improvements in therapeutic methods, such as neurosurgical resection with combined radio-chemotherapy, patients suffering from GBM still have a short median survival time about 15 months [4–6]. Poor survival of GBM patients is considered in large proportion to be due to complicated immunosuppressive microenvironment [7], the infiltrative character [8] and microscopic diffusion [9] of GBM cells, and distinct metabolic mechanisms [10]. With the prominent progress in comprehending the molecular mechanisms of GBM, researchers have stepped into some novel fields to investigate related treatment modalities [11, 12]. However, many therapies failed to show a promising effect due to the complexity of GBM microenvironment [13, 14], the blood-brain barrier (BBB) and rapid drug resistance [13]. Compared to the limited success of conventional therapies, immunotherapy seems to be an appealing strategy, utilizing immune system to attack cancer cells [15, 16]. Immune checkpoints, defined as regulatory molecules that participate in T cell related immune response and immune system self-tolerance [17, 18], also play a critical role in the development of GBM. Given immunosuppression and immune evasion impede the success of immunotherapeutic strategies [19], further exploration is still required for immunotherapy as a new treatment strategy.

PDIA3 (also known as ERp57), an important member of the protein disulfide isomerase (PDI) family, has drawn the attention of researchers to explore its role in human cancers due to its widespread implication in disease development. PDIA3 has been identified as a potential target in indirect regulation of epidermal growth factor receptor (EGFR) [20], association with the major histocompatibility complex (MHC) peptide-loading complex (PLC) [21], binding calreticulin (CRT) [22], and mechanistic/mammalian target of rapamycin (mTOR) signaling [23]. Due to the negative influence on antigen presentation and MHC PLC stability, downregulated MHC class I molecules facilitated tumor cells to avoid immune surveillance, which was also related to PDIA3 overexpression [21, 24, 25]. Moreover, PDIA3 is necessary to regulate the exposure of CRT on the cell surface for immune cell death [22]. In epithelial ovarian cancer, increased PDIA3 has been linked to low expression of Dicer, which is a marker of metastasis and poor prognosis [26]. In colon cancer, PDIA3 is also responsible for an efficient and specific T-cell response [27]. Besides, in diffuse gliomas, high expression of PDIA3 has an influence on glioma progression and predicts worse survival outcome and therapeutic response of glioma patients [28]. These findings supported that PDIA3 might have an remarkable influence on immunogenicity and invasion of cancer cells.

To clarify a potential role of PDIA3 in all gliomas, here, we performed analysis based on publicly available databases. Our findings were verified in TCGA and CGGA datasets. PDIA3 was found to be upregulated in gliomas and related to suppressive tumor microenvironment, indicating that PDIA3 might be a potential prognostic biomarker or therapy target in the clinical treatment of gliomas.

## RESULTS

### Associations of PDIA3 expression with clinical and molecular characteristics in gliomas

**Figure 1:**
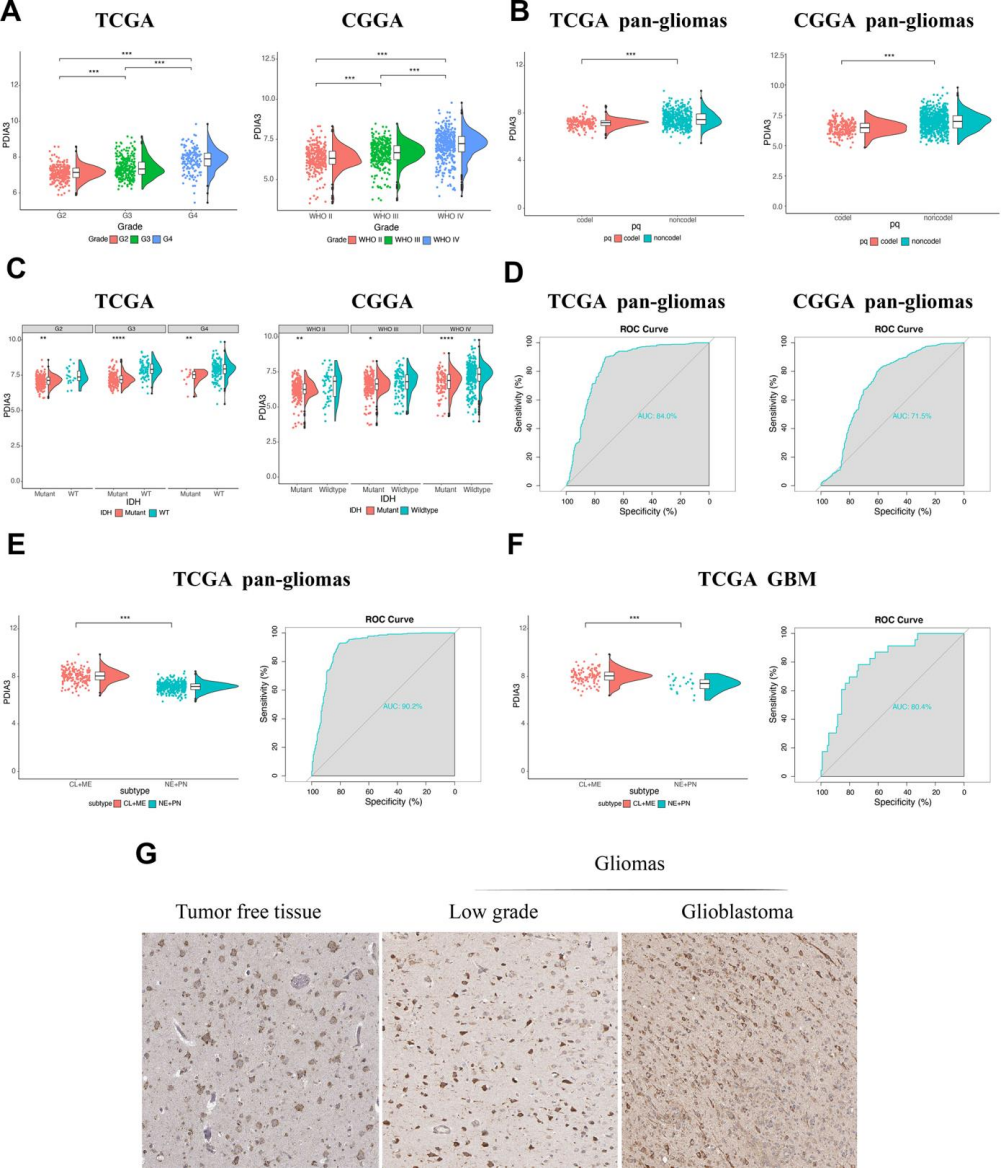
**The relationship between PDIA3 expression and various characteristics.** (**A**) PDIA3 expression in different WHO grades from TCGA and CGGA. (**B**) PDIA3 was upregulated in 1p/19q non-codeletion cases. (**C**) PDIA3 expression in different IDH state from TCGA and CGGA dataset. (**D**) ROC curve analysis showed that PDIA3 had 84.0% and 71.5% sensitivity and specificity to predict IDH wild-type state gliomas in TCGA and CGGA, respectively. (**E**) The PDIA3 expression pattern in pan-gliomas based on TCGA molecular subtypes. ROC curves showed PDIA3 as a predictor of more aggressive subtype gliomas. (**F**) The PDIA3 expression pattern in GBM from the TCGA molecular subtype. ROC curve analysis showed that PDIA3 had 80.4% sensitivity and specificity to predict more aggressive subtype gliomas. (**G**) PDIA3 has higher expression in GBM than in LGG at the protein level. *P < .05, **P < .01, ***P < .001.

Based on expression data from TCGA and CGGA datasets, PDIA3 was found to be highly correlated with U87 cell lineage (Supplementary Figure 1A) and various tumors, including GBM and low grade glioma (LGG) (Supplementary Figure 1B). Although there was no significant difference among complete remission, partial remission, and stable disease in regard to PDIA3 expression, PDIA3 had higher expression in progressive disease than in complete remission (Supplementary Figure 1C). And based on World Health Organization (WHO) classification, PDIA3 had the highest expression in GBM (WHO grade IV) compared to LGG samples (WHO grade II and grade III) (Figure 1A). PDIA3 expression was enriched in higher histopathologic malignancies (Supplementary Figure 1D) and 1p/19q non-codeletion cases (Figure 1B). Furthermore, Some genetic alterations including IDH mutation and 1p/19q codeletion were significantly associated with heterogeneous tumor histology based on 2016 WHO classification [29]. In our study, PDIA3 expression was observed in IDH wild-type gliomas of different grades (Figure 1C). Additionally, ROC curve analysis showed that PDIA3 had 84.0% and 71.5% sensitivity and specificity to predict IDH wild-type state gliomas in TCGA and CGGA, respectively(Figure 1D), which indicated worse outcome in the progression of glioma [30]. Except genetic alternations, epigenetic alterations including DNA methylation equally facilitate carcinogenesis [31]. PDIA3 was positively associated with MGMT unmethylated samples in pan-gliomas in TCGA, but there was no significant association in CGGA (Supplementary Figure 2A). Besides, among 5 methylation probes designed for PDIA3 from TCGA, all of them exhibited negative association with expression of PDIA3 and correlation was not prominent, which only 2 of them showed statistically significant association with expression of PDIA3 (Supplementary Figure 2B–2F). These findings indicated that the upregulated expression of PDIA3 correlated with a more malignant phenotype of glioma.

Subsequently, we evaluated inter-tumor and intra-tumor heterogeneous characteristics of PDIA3 in gliomas. Based on transcriptomic and genomic dimensions, molecular classification of human gliomas was divided into several subtypes, including classical (CL), mesenchymal (ME), pro-neural (PN), and neural (NE) [32]. CL and ME subtypes are more aggressive compared to PN or NE subtypes [33]. As shown in Figure 1E, increased PDIA3 expression was more correlated with the CL and ME subtypes than PN and NE subtypes in pan-glioma samples. Meanwhile, the area under the curve (AUC) was 80.4% with regard to CL and ME subtypes in GBM samples (Figure 1F), indicating that PDIA3 served as an effective predictor for CL and ME subtypes.

Then we also quantified the intra-tumor expression of PDIA3. Radiographically, the contrast-enhanced (CE) GBM area has different components compared with the non-contrast-enhanced (NCE; abnormal T2/FLAIR signal) GBM edge, which indicated that tumor cells infiltrated these edema tissues. Our analysis revealed that PDIA3 was highly enriched in CE regions compared with NCE or normal tissues (Supplementary Figure 1E). Based on the Ivy Glioblastoma Atlas Project, high expression of PDIA3 was abundant in hyperplastic blood vessels, microvascular proliferation and peri-necrotic zones (Supplementary Figure 1F), serving as a crucial role in the progression of tumors. To further confirm the upregulation of PDIA3 expression at the protein level, we downloaded the results of IHC staining for PDIA3 from the The Human Protein Atlas (https://www.proteinatlas.org) (Figure 1G). PDIA3 has higher expression in LGG and GBM compared to normal brain tissue, which the expression of PDIA3 is also higher in GBM than LGG. Taken together, these results suggested that PDIA3 might be the predictor for a more aggressive subtype in gliomas and play an important role in the progression of gliomas.

### PDIA3 expression is relevant to worse survival in gliomas

**Figure 2:**
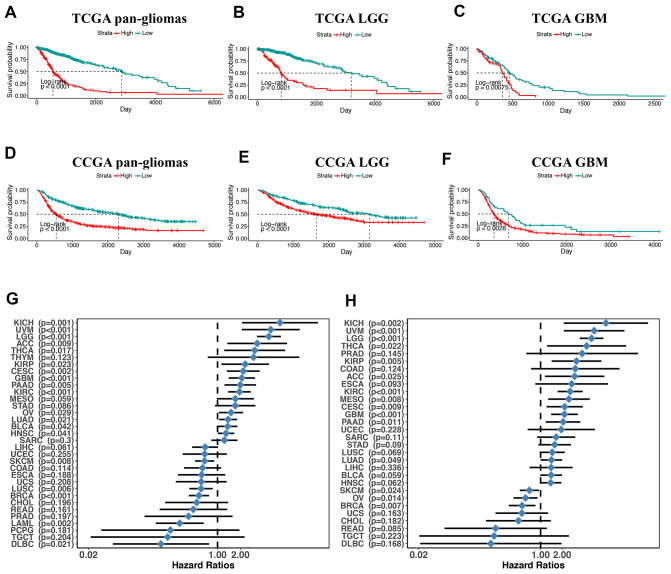
**overall survival in glioma patients with low and high PDIA3 expression.** Kaplan – Meier analysis of overall survival was performed in pan-glioma (**A**, **D**), LGG (**B**, **E**) and GBM (**C**, **F**) patients based on TCGA and CGGA datasets. (**G**) Subgroup analyses estimating prognostic value of PDIA3 in OS in different cancer types from TCGA. The length of horizontal line represents the 95% confidence interval for each group. The vertical dotted line represents the HR of all patients. HR < 1.0 indicates that high TMEscore is a favorable prognostic biomarker. Number of patients is indicated. (**H**). Subgroup analyses estimating prognostic value of PDIA3 in DSS in different cancer types from TCGA. The length of horizontal line represents the 95% confidence interval for each group. The vertical dotted line represents the HR of all patients. HR < 1.0 indicates that high TMEscore is a favorable prognostic biomarker. Number of patients is indicated. KICH, Kidney Chromophobe; UVM, Uveal Melanoma; LGG, Brain Lower Grade Glioma; ACC, Adrenocortical carcinoma; THCA, Thyroid carcinoma; THYM, Thymoma; KIRP, Kidney renal papillary cell carcinoma; CESC, Cervical squamous cell carcinoma and endocervical adenocarcinoma; GBM, Glioblastoma multiforme; PAAD, Pancreatic adenocarcinoma; KIRC, Kidney renal clear cell carcinoma; MESO, Mesothelioma; STAD, Stomach adenocarcinoma; OV, Ovarian serous cystadenocarcinoma; LUAD, Lung adenocarcinoma; BLCA, Bladder Urothelial Carcinoma; HNSC, Head and Neck squamous cell carcinoma; SARC, Sarcoma; LIHC, Liver hepatocellular carcinoma; UCEC, Uterine Corpus Endometrial Carcinoma; SKCM, Skin Cutaneous Melanoma; COAD, Colon adenocarcinoma; ESCA, Esophageal carcinoma; UCS, Uterine Carcinosarcoma; LUSC, Lung squamous cell carcinoma; BRCA, Breast invasive carcinoma; CHOL, Cholangiocarcinoma; READ, Rectum adenocarcinoma; PRAD, Prostate adenocarcinoma; LAML, Acute Myeloid Leukemia; PCPG, Pheochromocytoma and Paraganglioma; TGCT, Testicular Germ Cell Tumors; DLBC, Lymphoid Neoplasm Diffuse Large B-cell Lymphoma. P-values were obtained from the log-rank test.

Next, we utilized Kaplan-Meier analysis to investigate the prognostic value of PDIA3 expression in human gliomas. In pan-glioma analysis of both TCGA and CGGA datasets, the overall survival (OS) of patients with high PDIA3 expression was significantly lower than that of patients with low PDIA3 expression (Figure 2A, 2D). Similar results were also observed in LGG and GBM patients (Figure 2). In addition, higher PDIA3 expression was associated with worse progression-free survival (PFS) and disease-specific survival (DSS) among pan-glioma patients, LGG, and GBM patients (Supplementary Figure 3). In pan-cancer analysis, PDIA3 indicated worse OS and DSS in adrenocortical carcinoma (ACC), breast invasive carcinoma (BRCA), kidney Chromophobe (KICH), kidney renal clear cell carcinoma (KIRC), kidney renal papillary cell carcinoma (KIRP), lung adenocarcinoma (LUAD), ovarian serous cystadenocarcinoma (OV), pancreatic adenocarcinoma (PAAD), skin Cutaneous Melanoma (SKCM), thyroid carcinoma (THCA), and uveal Melanoma (UVM) (Supplementary Figures 4, 5). The prognostic value of the PDIA3 in pan-cancer was also assessed. The heterogeneous results indicated that the PDIA3 was a hazardous prognostic marker in eighteen independent tumor cohorts and a favorable prognostic marker in fifteen independent tumor cohorts including melanoma in regard to OS (Figure 2G). Similar results were observed in the analysis in regard to DSS (Figure 2H). These results revealed that PDIA3 might serve as a biomarker to predict the poor prognosis in gliomas.

### PDIA3 expression is associated with distinct patterns of genomic alterations

**Figure 3:**
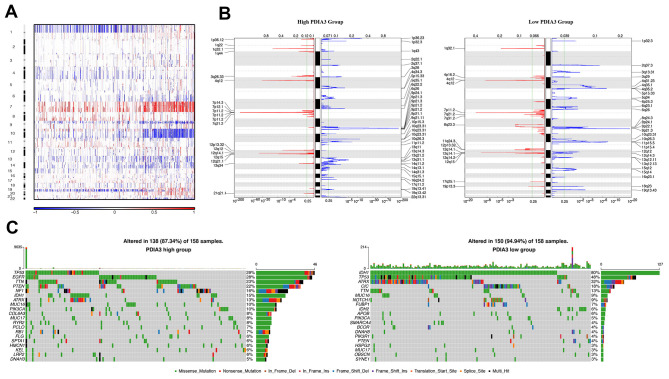
**Distinct genomic profiles correlated with PDIA3 expression.** (**A**) The overall CNAs profile arranged by high and low PDIA3 expression. Blue (deletion); red (amplification). (**B**) Frequency of amplifications and deletions in gliomas. Deletion is blue and amplification is red. (**C**) Distinct somatic mutations in gliomas.

In order to figure out the association between PDIA3 expression levels and gliomas genomic profiles, CNA and somatic mutation analysis were performed in TCGA dataset. A global CNA profile was observed from the comparison of clusters with low PDIA3 expression (n = 158) and high PDIA3 expression (n = 158) (Figure 3A, 3B). The high PDIA3 expression cluster frequently showed amplification of chr7 and deletion of chr10, both of which were typical genomic events in GBM (Figure 3A); while deletion of 1p and 19q, a genomic hallmark of oligodendroglioma, occurred more frequently in PDIA3 low cluster (Figure 3B). In high PDIA3 expression samples, frequently amplified genomic regions, containing oncogenic driver genes such as PDGFRA (4q12), EGFR (7p11.2) and CDK4 (12q14.1), were accompanied by CDKN2A/CDKN2B (9p21.3) and PTEN (10q23.3) deletion peaks. Furthermore, analysis of somatic mutation profiles indicated a high frequency of mutations in EGFR (28%), TTN (23%), PTEN (22%) and NF1 (16%) in the high PDIA3 expression cluster, while IDH1 (80%), ATRX (32%), CIC (18%) were more frequently mutated in the PDIA3 low expression cluster (Figure 3C).

Due to the location of PDIA3 gene on 15q15.3, a chromosomal band lost in parts of gliomas [34–36], we further explored the association between CN and PDIA3 expression. In both pan-gliomas and GBMs, tumors with PDIA3 CN loss expressed significantly lower levels of PDIA3 mRNA (Supplementary Figure 6). Thus, these results showed that PDIA3 expression could be altered by gross chromosomal changes in human gliomas.

### PDIA3 is related to infiltrating immune and stromal cells in the gliomas microenvironment

**Figure 4:**
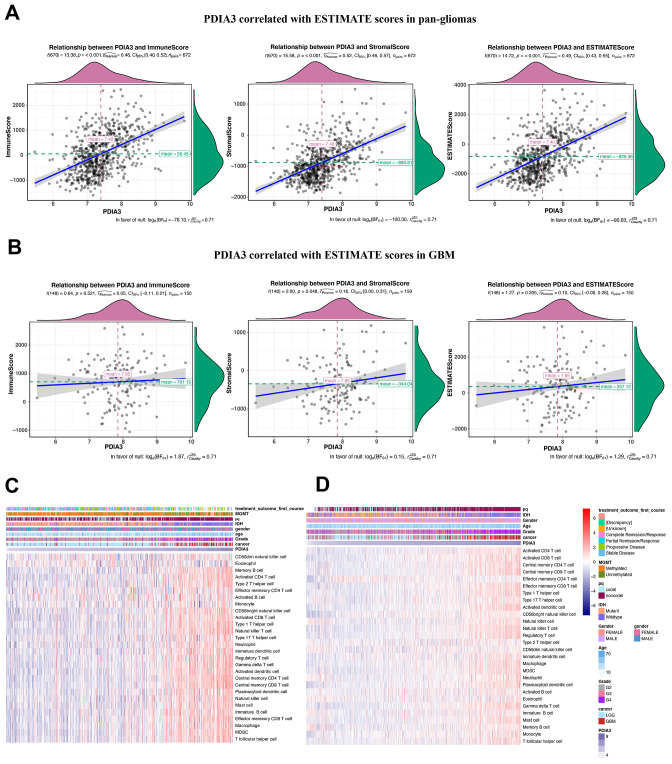
**Relationship between PDIA3 expression and ESTIMATE scores in gliomas. PDIA3 expression was positively associated with immune score, stromal score and ESTIMATE score in** (**A**) pan-glioma and (**B**). GBM patients. Heatmaps illustrate PDIA3 related specific infiltrating cell types based on (**C**). TCGA and (**D**). CGGA pan-glioma data.

Based on molecular studies, major cell components of tumors, such as infiltrating stromal and immune cells are responsible for cancer biology and tumor signaling [37]. Thus, we performed an analysis to identify the relationship between PDIA3 and ESTIMATE scores. We revealed that PDIA3 was positively related to immune score, stromal score, and ESTIMATE score in pan-gliomas with significant trends (Figure 4A) and in GBM samples with insignificant trends (Figure 4B), which indicated that PDIA3 was involved in immune and stromal cells infiltration. In order to elucidate specific cell types that might be affected by PDIA3 in tumor microenvironment, we used cell type enrichment analysis to study the association between PDIA3 and 28 populations of immune cells and stromal cells to further explore the role of elevated PDIA3 in gliomas [38]. A positive association was observed between PDIA3 expression and various infiltrating immune cell types, which consisted of cells responsible for anti-tumor response such as NK cells, CD4+ T effector memory cells (TEM), CD8+ TEM, and immunosuppressive cells such as DCs, MDSCs, regulatory T cells (Treg), macrophages, mast cells, neutrophils, monocytes (Figure 4C, 4D). Additionally, in the 10-immune cell lineage analysis, high PDIA3 expression gliomas were highly correlated with several stromal cells, including epithelial cells, astrocytes and fibroblasts [39] (Supplementary Figure 7A–7D). Thus, our findings supported that PDIA3 might be involved in the infiltration of immune and stromal cells in gliomas microenvironment.

### Single cell sequencing revealed the cell clusters correlated with PDIA3

**Figure 5:**
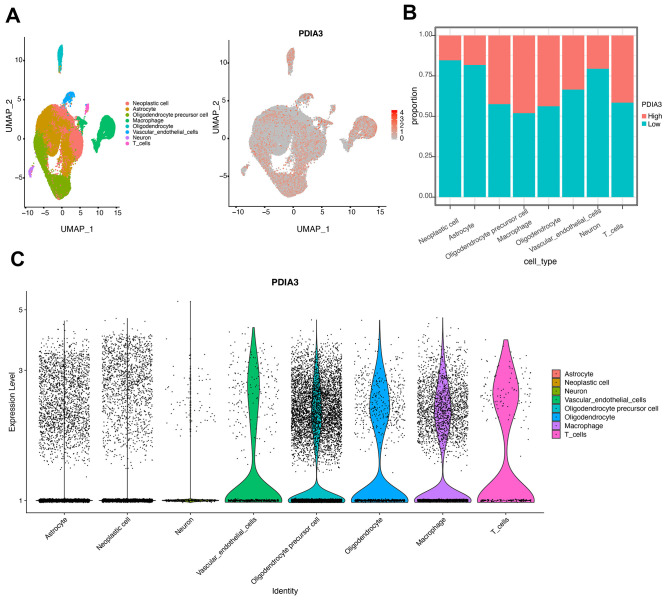
**Identification of cell types in single cell sequencing analysis.** (**A**) UMAP plot of tumor cells showing eight clusters with patient effects regressed out. Gray area represents the whole cell clusters. The red dot represents cell with PDIA3 expression. (**B**) The proportion of cells with PDIA3^high^ or PDIA3^low^ expression in eight cell clusters. (**C**) The expression level of PDIA3 in eight cell clusters.

We also performed the single cell data sequencing analysis. After clustering all the tumor cells with patient effects regressed out, we identified eight clusters of cells (Figure 5A), Neoplastic cell, Astrocyte, Oligodendrocyte precursor cell, Macrophage, Oligodendrocyte, Vascular endothelial cells, Neuron, and T cells, all of which come from all eight glioma patients. The expression landscape of PDIA3 in all eight clusters of cells was also shown in Figure 5A. We found that high PDIA3 expression occupied the relatively highest proportion in macrophage and T cells (Figure 5B). Moreover, the exact expression level of PDIA3 was visualized in Figure 5C, which further confirmed that PDIA3 was highly correlated with macrophage and T cells.

### PDIA3 is associated with T cell immunity in gliomas

**Figure 6:**
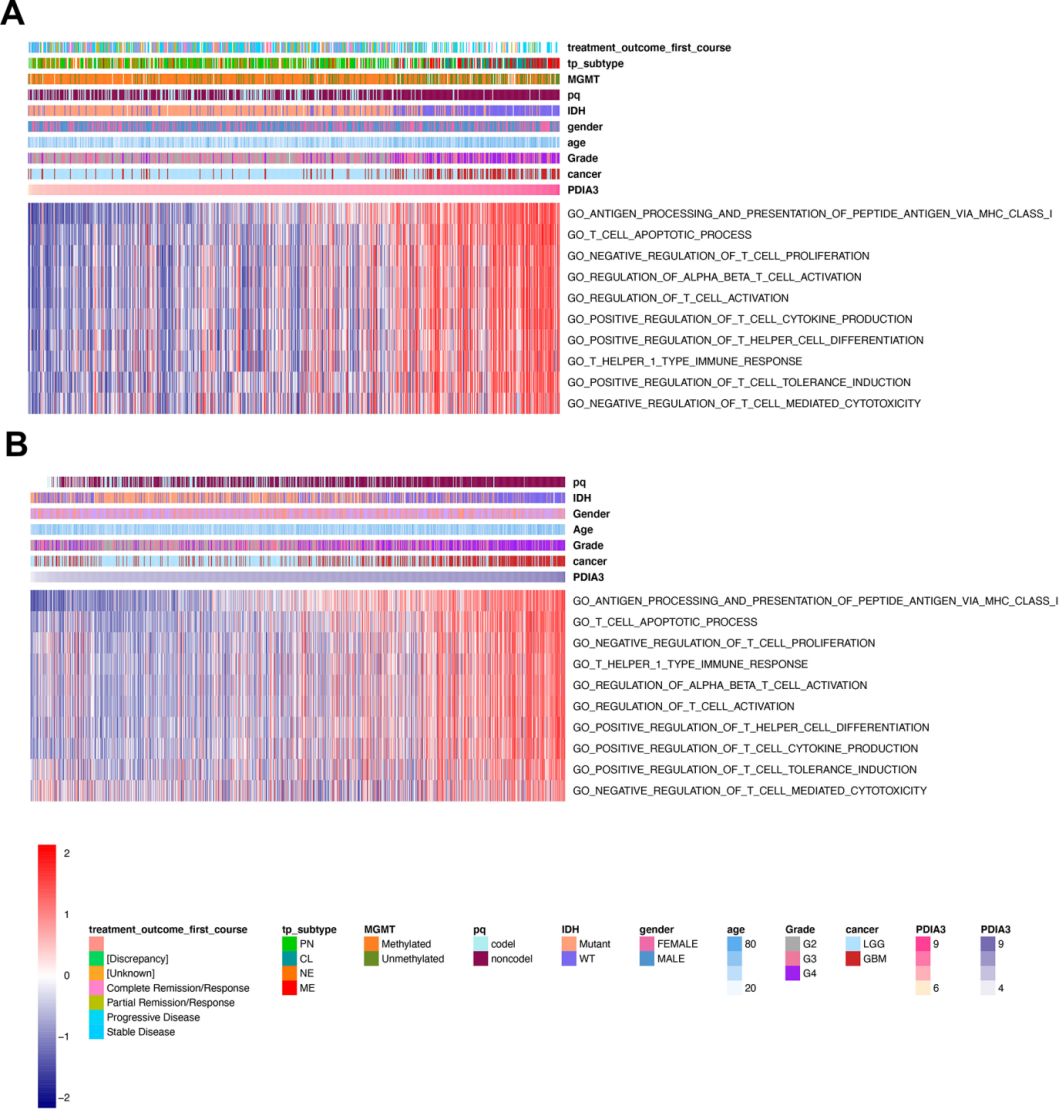
**PDIA3 related to T cell immunity in gliomas.** The association between PDIA3 and T cell related immune response from (**A**) TCGA and (**B**). CGGA pan-glioma data

Several studies have demonstrated that PDIA3 might play an important role in facilitating the efficacy of T cell-based immunotherapies [27, 40, 41]. To confirm whether PDIA3 was involved in T cell immunity, we performed GSVA analysis in TCGA cohort and found that PDIA3 was correlated with negative regulation of T cell mediated cytotoxicity and negative regulation of T cell proliferation (Figure 6A). On the contrary, PDIA3 was positively correlated with antigen processing and presentation of peptide antigen via MHC class I molecules, regulation of alpha beta T cell activation, regulation of T cell activation, positive regulation of T cell tolerance induction, positive regulation of T cell cytokine production, T helper 1 type immune response, and positive regulation of T helper cell differentiation. These results was subsequently verified in CGGA cohorts (Figure 6B). Thus, PDIA3 might serve as a crucial mediator in suppressing T cell related anti-tumor immune response in the glioma microenvironment.

### PDIA3 is correlated with other immune checkpoint members in gliomas

**Figure 7:**
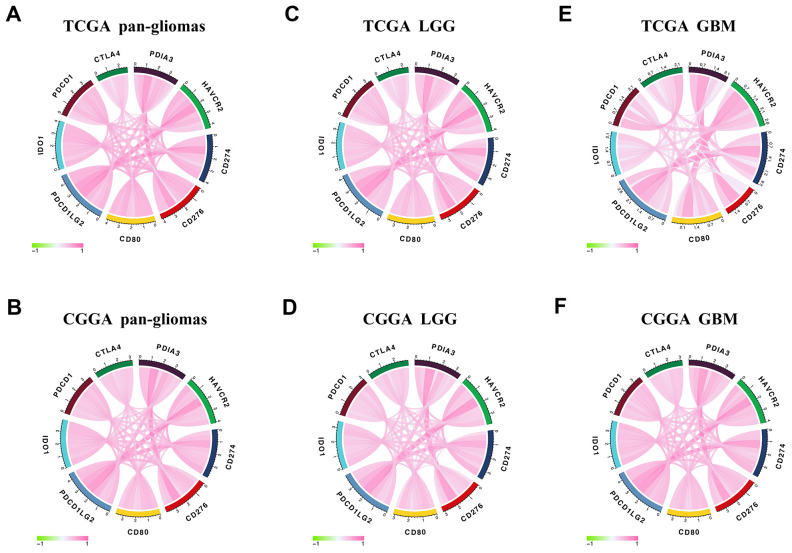
**Correlation between PDIA3 and immune checkpoint members.** PDIA3 is relevant to other immune checkpoint molecules in pan-gliomas (**A**, **B**), LGG (**C**, **D**), and GBM (**E**, **F**) from TCGA (upper row) and CGGA (lower row) datasets.

In view of the increasing clinical benefits of targeting immune checkpoints as the combination therapy [42, 43], we enrolled several immune checkpoint molecules into correlation analysis to assess their relationship with PDIA3 in glioma samples. As shown in Figure 7, PDIA3 was highly positively correlated with HAVCR2, CD274, PDCD1LG2 and others in pan-gliomas and LGG and GBM samples, and PDIA3 demonstrated a high positive correlation with PDCD1LG2 in GBM. Thus, these results indicated that PDIA3 might serve as a potential molecule to modulate immunosuppression-related signaling pathways through the combination with other immune checkpoints in the glioma microenvironment.

### PDIA3 is involved in inflammatory activities in gliomas

**Figure 8:**
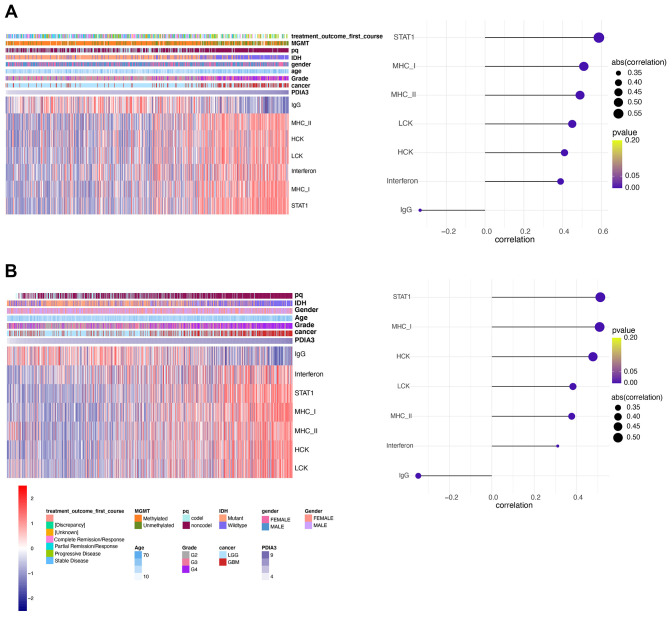
**PDIA3 correlates with inflammatory activities in gliomas.** Heatmaps illustrate PDIA3 related inflammatory activities in pan-gliomas in (**A**) TCGA and (**B**) CGGA datasets.

Since our analysis demonstrated that PDIA3 was correlated with inflammation in gliomas, we subsequently examined seven genes to identify the association of PDIA3 with these inflammatory activity signatures [44]. PDIA3 expression was positively correlated with HCK, LCK, MHC-I, MHC-II, STAT1, and interferon, but negatively related to IgG in pan-glioma (Figure 8A, 8B) and GBM (Supplementary Figure 8) analysis based on both TCGA and CGGA datasets. These data showed that PDIA3 was abundant in macrophages activation, signal transduction of T cells and antigen-presenting cells, while negatively interacted with B lymphocytes-related genes. Thus, we also confirmed that PDIA3 served as a pivotal molecule in immune and inflammatory progression of gliomas.

## DISCUSSION

With the increasing attraction of PDIA3 in diverse cancers and immunotherapy, it is deemed as a potential molecule improving current therapeutic strategies. Since the status of PDIA3 in glioma samples and the association between PDIA3 and immune responses haven’t been clearly clarified, we investigated the landscape of PDIA3 among gliomas using a large-scale bioinformatic analysis. Here, our data indicated that the upregulated PDIA3 expression was highly correlated with GBM, especially in IDH wildtype status based on 2016 WHO classification. Besides, PDIA3 was highly enriched in CL and ME subtype gliomas and served as a sensitive diagnostic marker. ME subtype contained abundant immunosuppressive cytokines and immune checkpoint inhibitors, presenting immunosuppression and aggression [32, 33, 45]. PDIA3 was also mostly localized in hyperplastic blood vessels, microvascular proliferation and peri-necrotic zones. And methylation at cg12737574 and cg17847446 might be the potential regulatory element at higher PDIA3 expression levels in GBM. However, the association between PDIA3 and MGMT is still unclear, which needs further exploration.

The PDIA3 gene with aberration has been observed in various tumors. Overexpression of PDIA3 is relevant to worse prognosis in cancers including laryngeal cancer [46], breast cancer [20, 47], cervical cancer [48], and epithelial ovarian cancer [26]. Critically, our findings also showed that high expression of PDIA3 was correlated with worse survival in TCGA and CGGA database. PDIA3 was actively involved in some processes facilitating immunosuppression and poor prognosis of glioma patients, including T cell tolerance induction and regulatory T cell differentiation [19, 49]. These results identified that PDIA3 widely participated in oncogenic processes and specifically predicted worse prognosis for glioma patients.

In the analysis of distinct genomic alternations, we observed that PDIA3 expression was positively relevant to somatic mutations and CNAs. In high PDIA3 expression samples, frequently amplified genomic peaks were detected in oncogenic drivers, including PDGFRA, EGFR and CDK4; in addition, tumor suppressor genes, such as CDKN2A/CDKN2B and PTEN were observed a deletion peak. Importantly genomic alternations and heterogeneity exerted a positive influence on tumor microenvironment transformation, tumor progression, and treatment resistance [50]. These results suggested that the expression of PDIA3 was related to malignant biological processes. Since immune checkpoint inhibitors have appeared potential benefits in cancer therapy, a combination of these inhibitors led to higher response rate and longer survival time in patients with melanoma and brain metastasis [17, 51, 52]. Our results showed that PDIA3 was highly correlated with HAVCR2, CD274, CD276, CD80, IDO1, PDCD1, CTLA-4, and PDCD1LG2 in pan-gliomas, LGGs, and GBMs. Therefore, it is suggested that the combination of PDIA3 and other immune checkpoint inhibitors might be a potential target for glioma therapy.

Previous studies have shown that PDIA3 was found to contribute to the breakdown of immune surveillance, tumor cell invasion, and immunologic cell death [21, 22, 24, 25]. In our study, we first explored the relationship between PDIA3 and ESTIMATE score, in which PDIA3 was positively associated with immune score, stromal score, and ESTIMATE score. Then, a specific analysis between PDIA3 and tumor microenvironment components showed that PDIA3 was positively associated with infiltrating immune and stromal cells, including DCs, MDSCs, TEM, Tregs, macrophages, mast cells, neutrophils, NK cells, monocytes. The single cell sequencing further confirmed that PDIA3 was correlated with macrophage and T cells. Moreover, GSVA analysis implied that PDIA3 suppressed T cell associated anti-tumour immune response. These results proved that PDIA3 had an impact on creating immunosuppressive microenvironment in gliomas. Compared with the results in previous researches, our study proved that PDIA3 upregulated MHC-I. Due to heterogeneity of each kind of tumor, microenvironment would be different. This discrepancy between gliomas and other tumors are potential direction in gliomas research field.

Taken together, we elaborated a potential role of PDIA3 as a molecular target in the anticancer therapies of gliomas based on our bioinformatics analysis. PDIA3 was positively associated with high malignancy of gliomas and worse survival of glioma patients. Meanwhile, PDIA3 was involved in inflammation, interaction with other immune checkpoint inhibitors, and suppression of anti-tumour immunity in the glioma microenvironment. Future studies are necessary to investigate the mechanism about the tumor microenvironment and immunoregulatory role of PDIA3.

## MATERIALS AND METHODS

### Data collection

This study was ethically approved by Xiangya Hospital, Central South University. From the The Cancer Genome Atlas (TCGA) and Chinese Glioma Genome Atlas (CGGA) datasets, we collected PDIA3 data from low grade glioma (LGG) and GBM samples. 672 samples from TCGA were downloaded from UCSC Xena (https://xenabrowser.net/). 1013 samples from CGGA were downloaded from CGGA website (http://www.cgga.org.cn/). RNA-seq data in regard to specific tumor anatomic structure in GBM was downloaded from Ivy Glioblastoma Atlas Project (http://glioblastoma.alleninstitute.org/). PDIA3 expression data in different radiographical regions of normal brain and GBM was downloaded from the Gill dataset. Immunohistochemical images with regard to PDIA3 were downloaded from https://www.proteinatlas.org. Single-cell expression matrices were obtained from the Gene Expression Omnibus (GEO; https://www.ncbi.nlm.nih.gov/) GSE138794 [53], which performed single-cell/nuclei RNA-sequencing of 28 gliomas. 8 scRNA sequeencing datasets including both LGG and high grade glioma (HGG) were used for analysis.

### Bioinformatic analysis

The cut-off point was calculated via the R package survminer in OS, PFS, DSS analyses. Correlation analysis of PDIA3 was performed using gene expression profiles from the TCGA and CGGA datasets with R language (https://www.r-project.org/). Somatic mutations and somatic copy number alternations (CNAs)which correspond to the cases with RNA-seq data, were downloaded from TCGA database. GSITIC analysis was adopted to determine the genomic event enrichment. CNAs associated with PDIA3 expression and the threshold copy number (CN) at alteration peaks were from GISTIC 2.0 analysis (https://gatkforums.broadinstitute.org). GSITIC analysis was performed based on the first 25% and last 25% of samples. The gene sets variation analysis (GSVA) package was used to analyze the differential expression in GO terms of immune related process and immune cell lineages from TCGA and CGGA samples. Correlation analysis was performed by the expression values of risk score and GO term, and the items with p<0.05 and high correlation coefficient (correlation coefficient >0.4) were selected. After Spearman correlation analysis, Heatmap was used to construct gene ontology (GO) analysis of the most correlated genes. ESTIMATE (Estimation of Stromal and Immune cells in Malignant Tumor tissues using Expression) algorithm was to evaluate the infiltration of immune cells and the presence of stromal cells in tumor samples, which generates three results including immune score (reflecting the level of immune cells infiltrations in tumor tissue), stromal score (reflecting the presence of stroma in tumor tissue), and estimate score (reflecting tumor purity). We processed the single-cell data expression matrix with the R package Seurat V3.1.2. Firstly, the single-cell gene expression data was normalized by 'NormalizeData', then the function "FindVariableGenes" was used to identify 2000 highly variable genes (HVGs). Next, we used "FindIntegrationAnchors" and "Integratedata" to merge 8 glioma sample datas [54]. After using "RunPCA" to perform principal component analysis (PCA), we constructed a K-nearest neighbor graph based on PCA via the "FindNeighbors" function and used the "FindClusters" to alternately combine cells together with the highest resolution. Finally, “UMAP” was used for visualization. In single cell sequencing analysis, the cut-off point was defined as the median value of all expression levels of PDIA3.

### Statistical analysis

Spearman correlation analysis was used to evaluate the correlations between continuous variables. The survival probability was described by Kaplan-Meier survival curves. The Student t-test was used to determine the expression levels of PDIA3 with regard to pathological characteristics. "SingleR" R package was used to identify the celltypes, we chose a glioma dataset in GEO(GSE84465) and datas in HumanPrimaryCell-AtlasData as a reference [55]. "VlnPlot" and "Feature-Plot" were used to visualize our gene expression. The linear relationship between gene expression levels was evaluated by the Pearson correlation. Survival package in R project was used for Cox regression analysis. All statistical analyses were performed using R project (version 3.4.1, https://www.r-project.org/). P-values or adjust P-values <0.05 were considered to be statistically significant. And all tests were two-sided.

### Availability of data and material

The datasets generated and analyzed during the current study are available in the Gene Expression Omnibus (https://www.ncbi.nlm.nih.gov/geo/), TCGA data source (https://xena.ucsc.edu) and CGGA data portal (http://www.cgga.org.cn).

## Supplementary Material

Supplementary Figures
