# Supplementary material for: PDIA3 correlates with clinical malignant features and immune signature in human gliomas
Source: Aging (Albany NY). 2020 Jul 19;12(15):15392–413. doi: 10.18632/aging.103601 (PMC7467394; doi:10.18632/aging.103601)
Supplement: Supplementary Figures [file aging-12-103601-s001..pdf]

SUPPLEMENTARY FIGURES

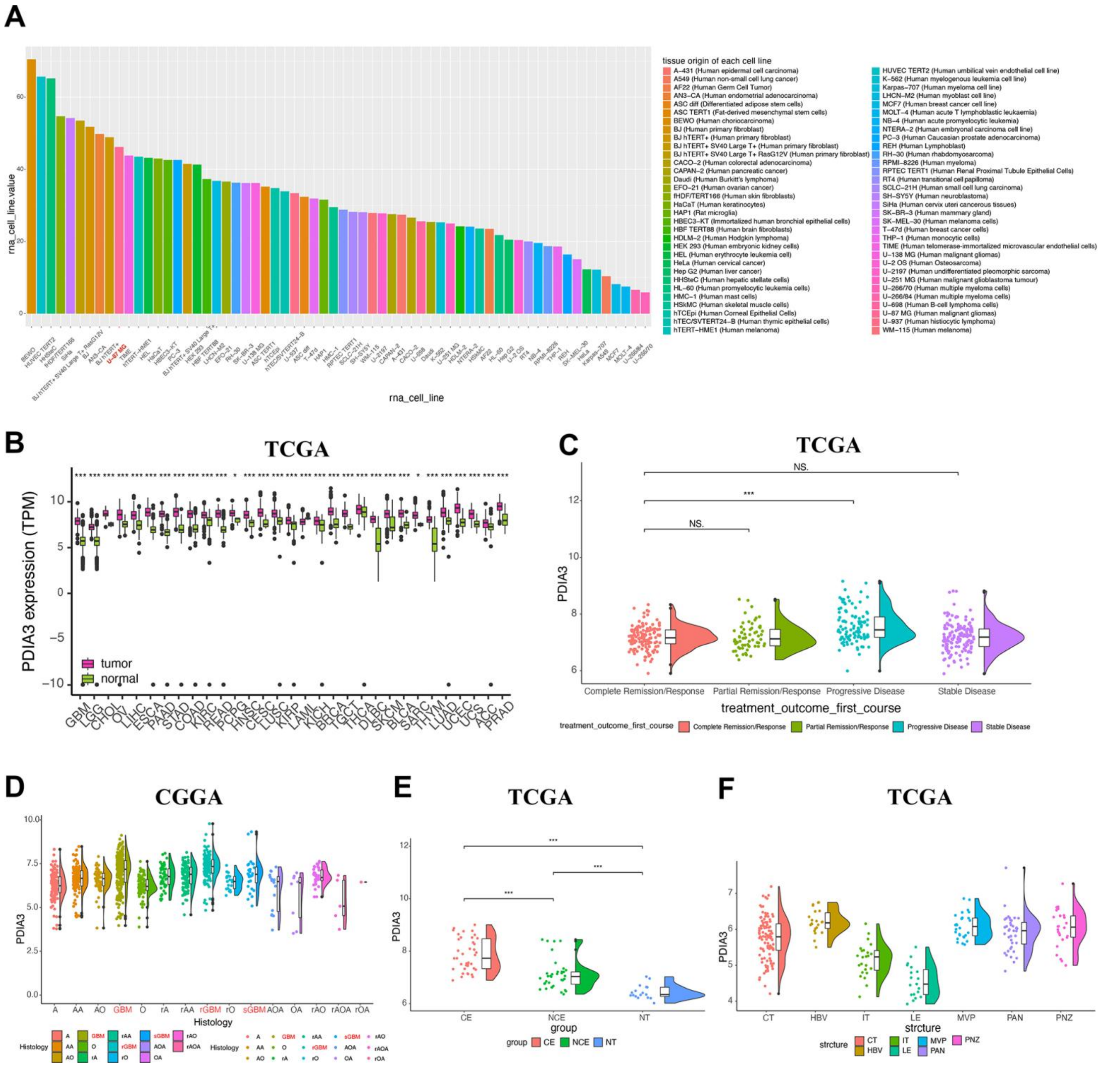

Supplementary Figure 1. clinical and molecular features in associations with PDIA3 expression. (**A**) PDIA3 expression in different cell lines. (**B**) PDIA3 expression in different tumors from TCGA dataset. GBM, Glioblastoma multiforme; LGG, Brain Lower Grade Glioma; CHOL, Cholangiocarcinoma; OV, Ovarian serous cystadenocarcinoma; LIHC, Liver hepatocellular carcinoma; ESCA, Esophageal carcinoma; PAAD, Pancreatic adenocarcinoma; STAD, Stomach adenocarcinoma; COAD, Colon adenocarcinoma; KIRC, Kidney renal clear cell carcinoma; READ, Rectum adenocarcinoma; PCPG, Pheochromocytoma and Paraganglioma; HNSC, Head and Neck squamous cell carcinoma; CESC, Cervical squamous cell carcinoma and endocervical adenocarcinoma; LUSC, Lung squamous cell carcinoma; KIRP, Kidney renal papillary cell carcinoma; LAML, Acute Myeloid Leukemia; KICH, Kidney Chromophobe; BRCA, Breast invasive carcinoma; TGCT, Testicular Germ Cell Tumors; THCA, Thyroid carcinoma; DLBC, Lymphoid Neoplasm Diffuse Large B-cell Lymphoma; SKCM, Skin Cutaneous Melanoma; BLCA, Bladder Urothelial Carcinoma; SARC, Sarcoma; THYM, Thymoma; LUAD, Lung adenocarcinoma; UCEC, Uterine Corpus Endometrial Carcinoma; UCS, Uterine Carcinosarcoma; ACC, Adrenocortical carcinoma; PRAD, Prostate adenocarcinoma. (**C**) PDIA3 expression in different treatment outcomes. (**D**). PDIA3 expression in different histopathologic classification from CGGA dataset. A, low-grade astrocytoma; AA, anaplastic astrocytoma; AO, anaplastic oligodendroglioma; GBM, glioblastoma; O, oligodendroglioma; rA, ; rA, recurrent low-grade

astrocytoma; rAA, recurrent anaplastic astrocytoma; rGBM, recurrent glioblastoma; rO, recurrent, oligodendroglioma; sGBM, secondary glioblastoma; AOA, anaplastic oligoastrocytoma; OA, oligoastrocytoma. (**E**) PDIA3 expression in different radiographical regions from TCGA dataset. CE, contrast-enhanced GBM; NCE, non-contrast enhanced GBM; NT, normal tissue. (**F**). Intra-tumor analysis of PDIA3 expression. LE (Leading Edge), IT (Infiltrating Tumour), CT (Cellular Tumour), PAN (Pseudopalisading Cells Around Necrosis), PNZ (Perinecrotic Zone), MVP (Microvascular Proliferation), and HBV (Hyperplastic Blood Vessels). NS, *, **, and *** indicate p < .05, P < .01, p < .001, and no significant difference, respectively.

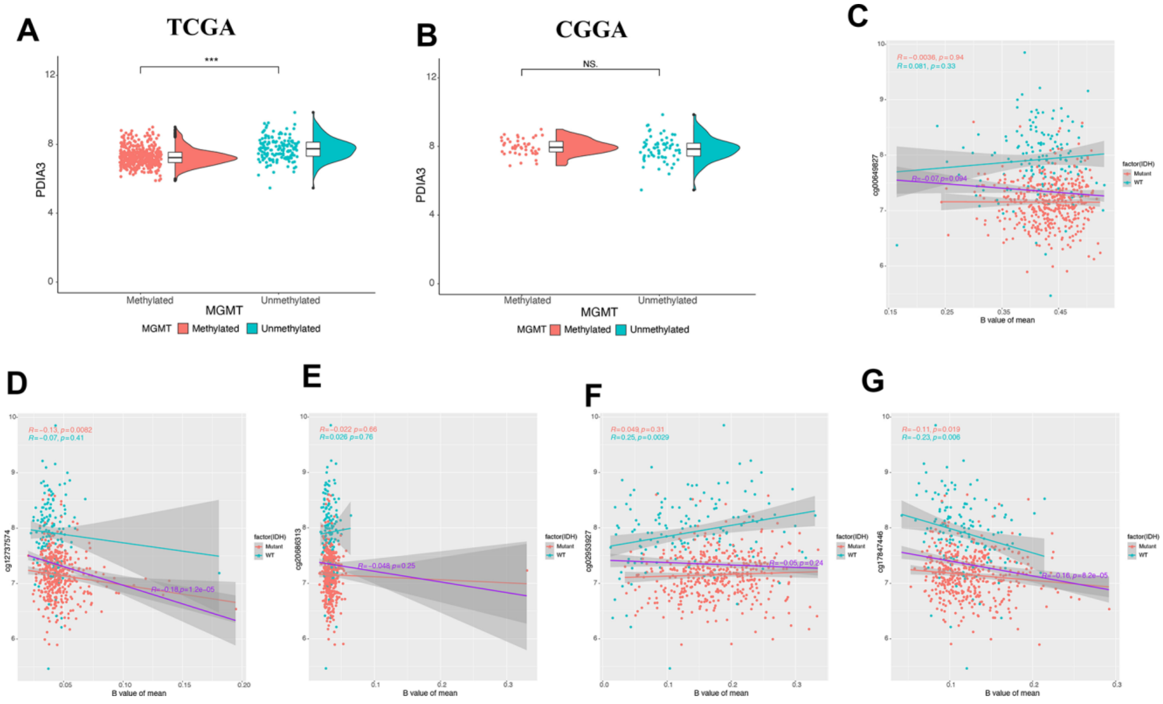

Supplementary Figure 2. Regulation of PDIA3 by methylation. Association between PDIA3 and MGMT methylation from (**A**) TCGA and (**B**) CGGA. Relationship between PDIA3 and methylation status at promoter region in TCGA: (**C**) cg00649827 loci; (**D**) cg12737574 loci; (**E**) cg20686313 loci; (**F**) cg02953927 loci; (**G**) cg17847446 loci. The orange dots indicate IDH-mutant samples, and cyan dots indicate IDH wild-type samples, respectively. The orange line and cyan line indicate linear regression between PDIA3 expression and promoter region methylation in IDH-mutant samples and IDH wild-type samples, respectively.

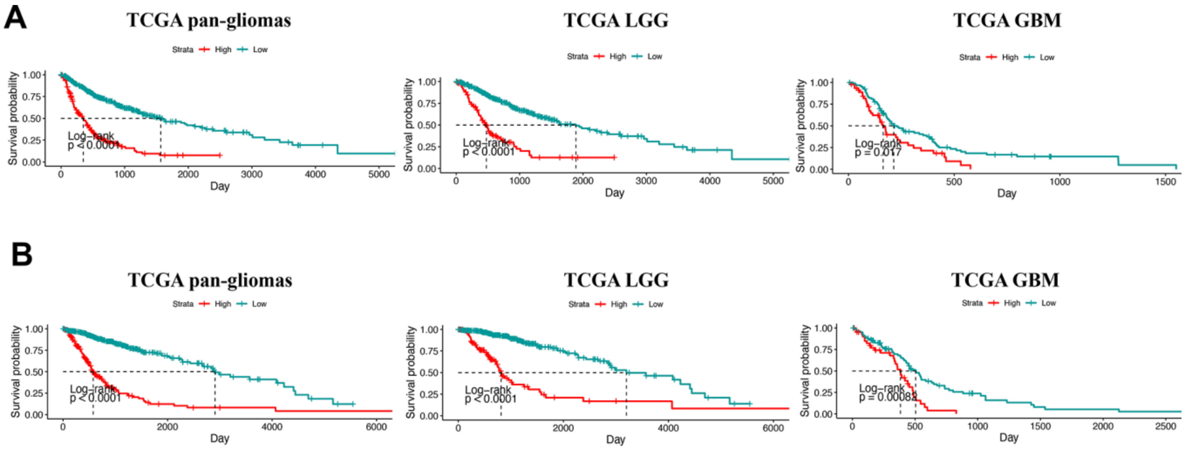

Supplementary Figure 3. Kaplan-Meier analysis using high vs low PDIA3 expression for (**A**) progression-free survival (PFS) in pan-glioma analysis, and LGG and GBM patients independently; (**B**). disease specific survival in pan-glioma analysis, and LGG and GBM patients independently. *P*-values were obtained from the log-rank test.

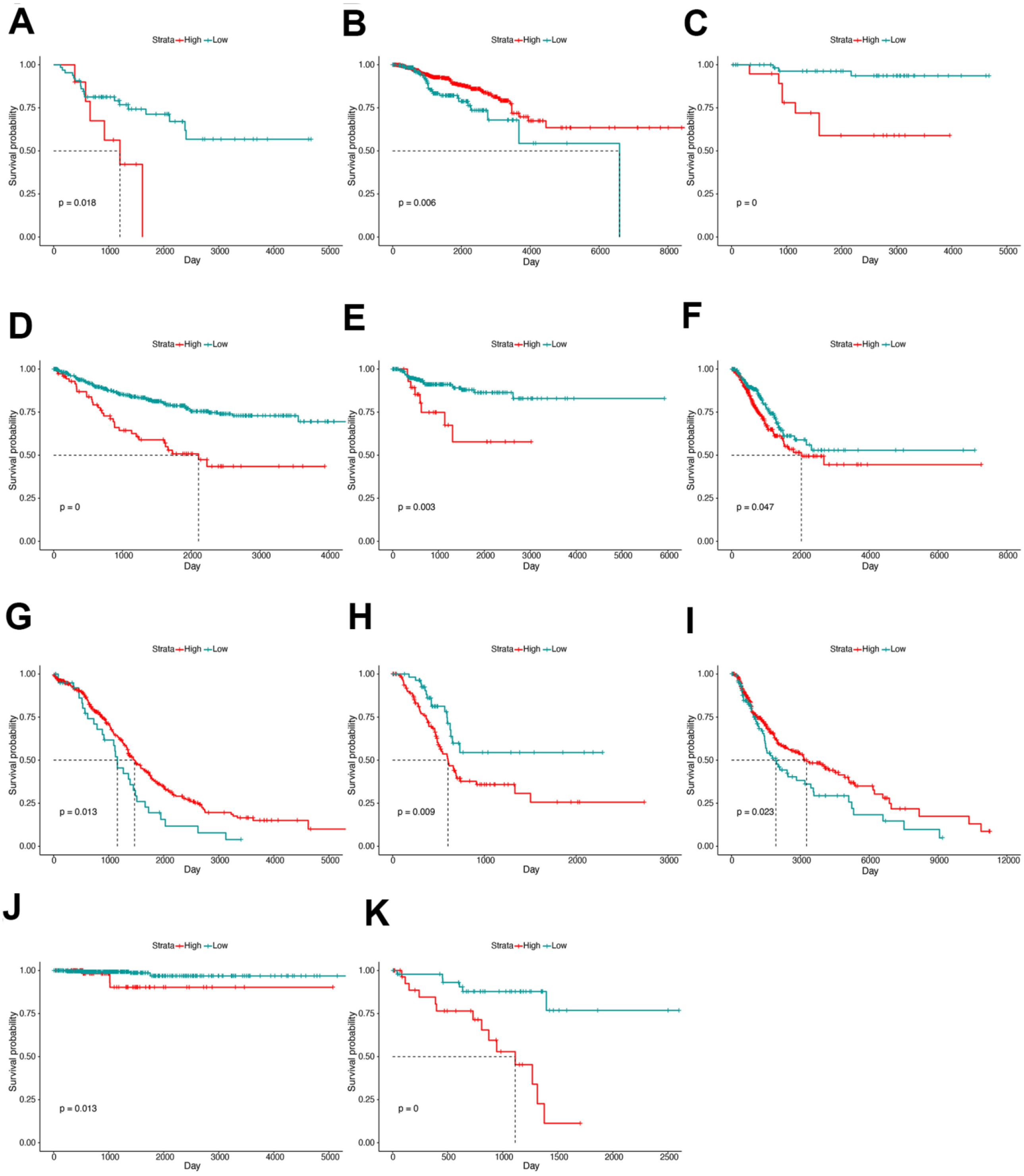

Supplementary Figure 4. Kaplan-Meier analysis using high vs low PDIA3 expression for disease specific survival (DSS) in different cancer. (**A**) ACC, Adrenocortical carcinoma; (**B**) BRCA, Breast invasive carcinoma; (**C**) KICH, Kidney Chromophobe; (**D**) KIRC, Kidney renal clear cell carcinoma; (**E**) KIRP, Kidney renal papillary cell carcinoma; (**F**) LUAD, Lung adenocarcinoma; (**G**). OV, Ovarian serous cystadenocarcinoma; (**H**) PAAD, Pancreatic adenocarcinoma; (**I**) SKCM, Skin Cutaneous Melanoma; (**J**) THCA, Thyroid carcinoma; (**K**) UVM, . *P*-values were obtained from the log-rank test.

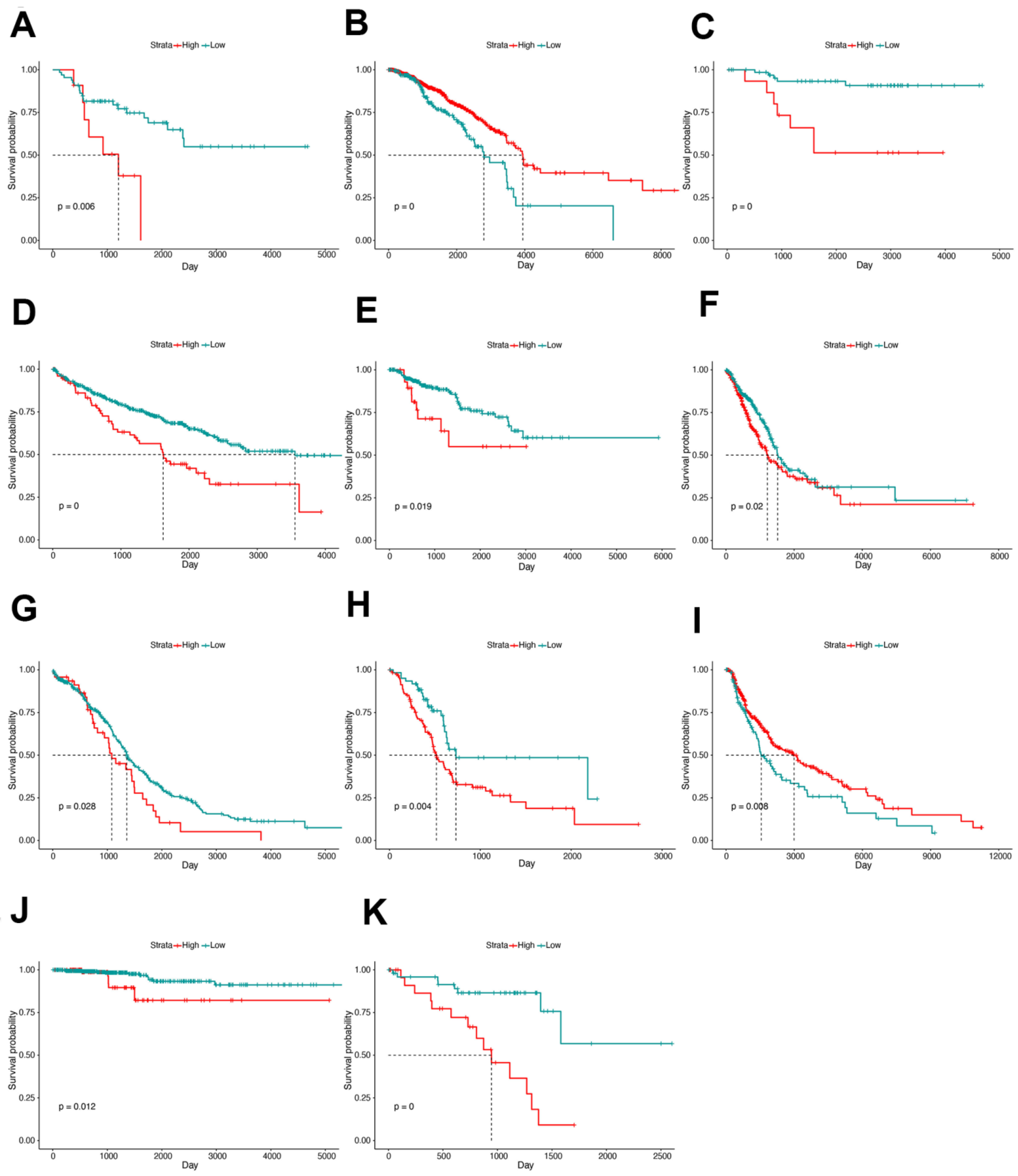

Supplementary Figure 5. Kaplan-Meier analysis using high vs low PDIA3 expression for pan-cancer survival in different cancer. (**A**) ACC, Adrenocortical carcinoma; (**B**) BRCA, Breast invasive carcinoma; (**C**) KICH, Kidney Chromophobe; (**D**) KIRC, Kidney renal clear cell carcinoma; (**E**) KIRP, Kidney renal papillary cell carcinoma; (**F**) LUAD, Lung adenocarcinoma; (**G**) OV, Ovarian serous cystadenocarcinoma; (**H**) PAAD, Pancreatic adenocarcinoma; (**I**). SKCM, Skin Cutaneous Melanoma; (**J**) THCA, Thyroid carcinoma; (**K**) UVM, . *P*-values were obtained from the log-rank test.

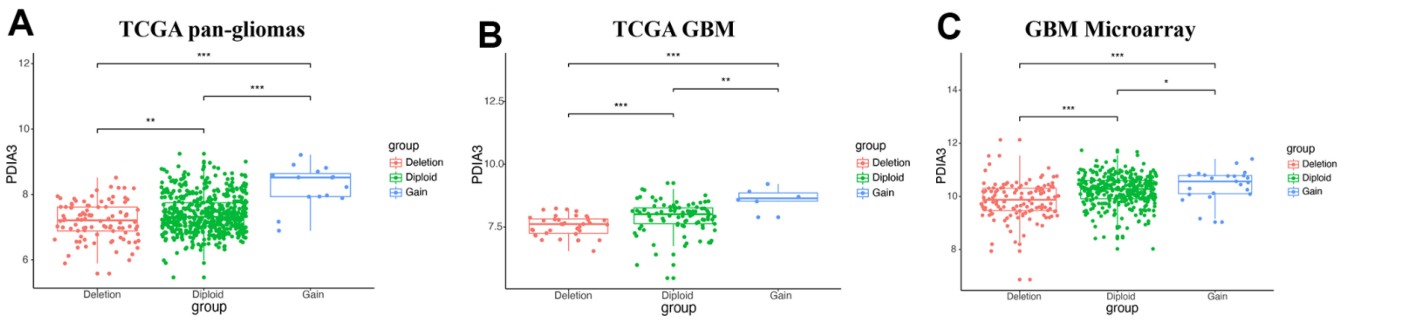

Supplementary Figure 6. Relationship between PDIA3 expression and PDIA3 copy number in (**A**) TCGA pan-gliomas, (**B**) TCGA GBM, and (**C**) GBM Microarray.

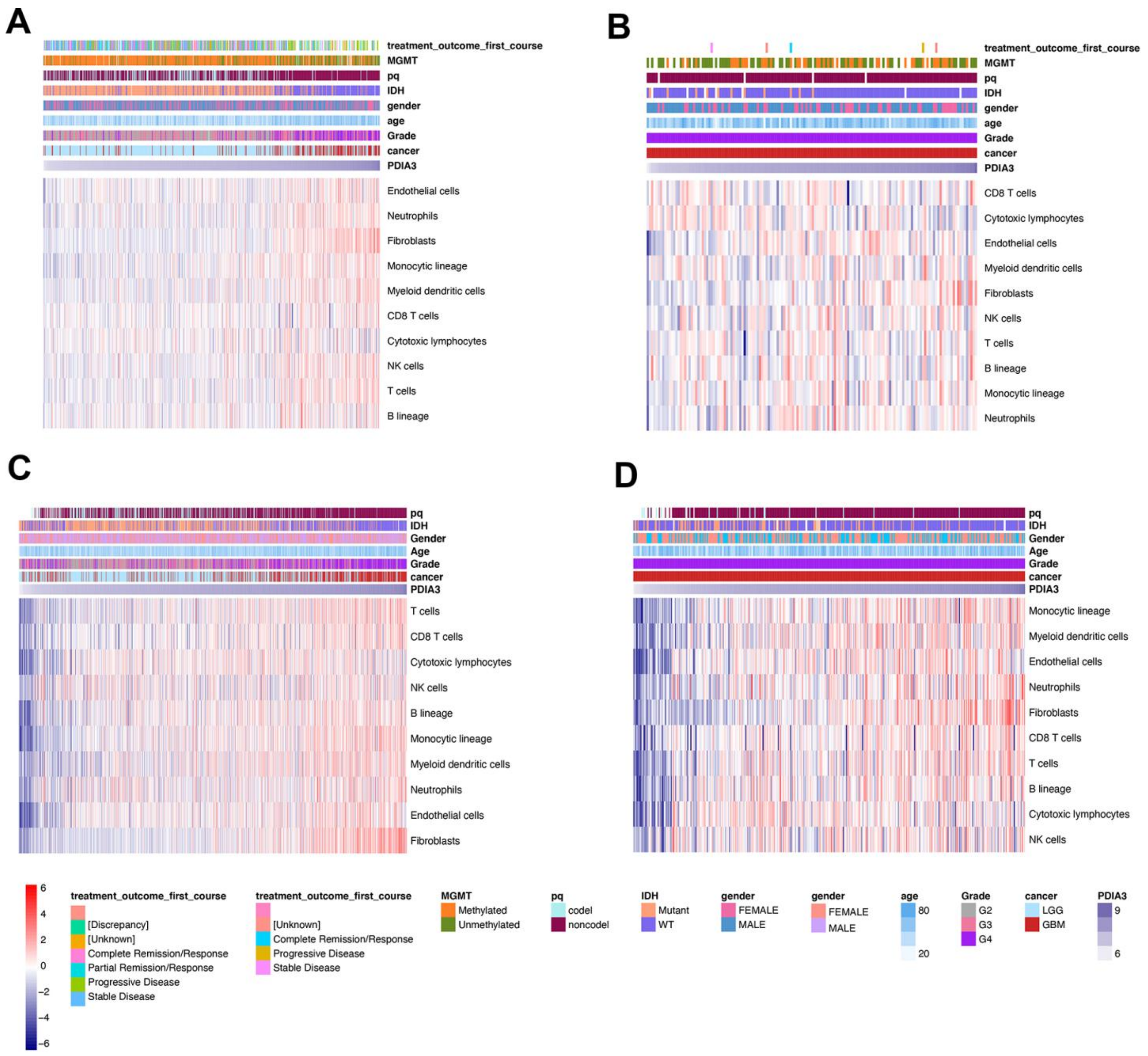

Supplementary Figure 7. Correlation of PDIA3 and 10-immune cell lineages genes in (**A**, **C**) pan-glioma analysis, and (**B**, **D**) GBM samples in TCGA (upper row) and CGGA (lower row) datasets. Expression values are z-transformed and are highlighted in red for high expression and blue for low expression, as indicated in the scale bar.

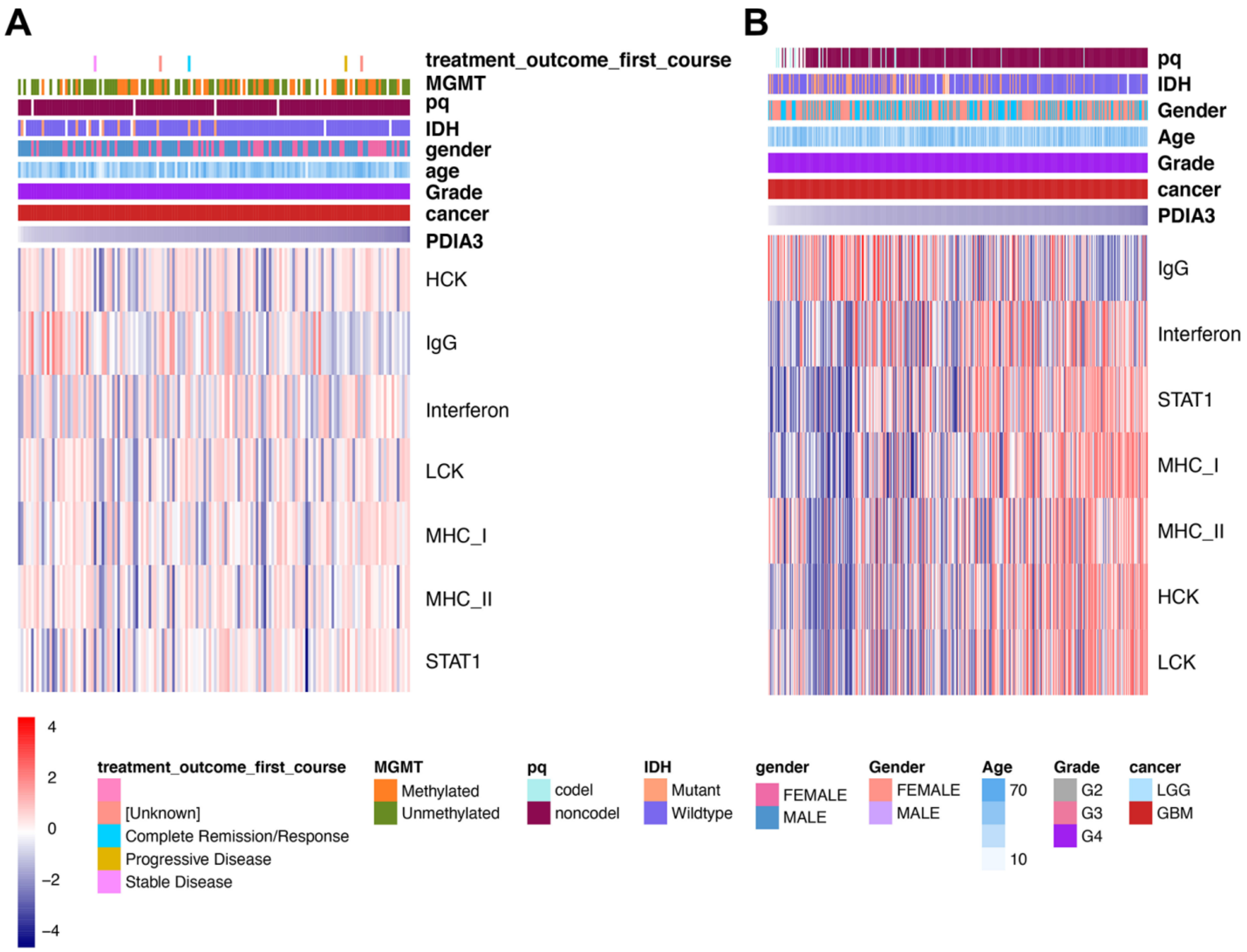

Supplementary Figure 8. Correlation of PDIA3 expression and inflammatory processes among GBM samples in (**A**). TCGA and (**B**) CGGA datasets. Expression values are z-transformed and are highlighted in red for high expression and blue for low expression, as indicated in the scale bar.
